# Genome-wide epistasis analysis reveals gene–gene interaction network on an intermediate endophenotype P-tau/Aβ_42_ ratio in ADNI cohort

**DOI:** 10.1038/s41598-024-54541-8

**Published:** 2024-02-17

**Authors:** Qiushi Zhang, Junfeng Liu, Hongwei Liu, Lang Ao, Yang Xi, Dandan Chen

**Affiliations:** 1 School of Computer Science, Northeast Electric Power University, 169 Changchun Street, Jilin, 132012 China; 2 School of Automation Engineering, Northeast Electric Power University, 169 Changchun Street, Jilin, 132012 China; 3 College of Intelligent Systems Science and Engineering, Harbin Engineering University, 145 Nantong Street, Harbin, China

**Keywords:** Epistasis, Quantitative trait, Alzheimer's disease, Alzheimer's disease, Epistasis, Quantitative trait, Alzheimer's disease, Alzheimer's disease

## Abstract

Alzheimer’s disease (AD) is a progressive neurodegenerative disorder and the most common cause of dementia in the elderly worldwide. The exact etiology of AD, particularly its genetic mechanisms, remains incompletely understood. Traditional genome-wide association studies (GWAS), which primarily focus on single-nucleotide polymorphisms (SNPs) with main effects, provide limited explanations for the “missing heritability” of AD, while there is growing evidence supporting the important role of epistasis. In this study, we performed a genome-wide SNP–SNP interaction detection using a linear regression model and employed multiple GPUs for parallel computing, significantly enhancing the speed of whole-genome analysis. The cerebrospinal fluid (CSF) phosphorylated tau (P-tau)/amyloid-$\beta _{42}$ (A$\beta _{42}$) ratio was used as a quantitative trait (QT) to enhance statistical power. Age, gender, and clinical diagnosis were included as covariates to control for potential non-genetic factors influencing AD. We identified 961 pairs of statistically significant SNP–SNP interactions, explaining a high-level variance of P-tau/A$\beta _{42}$ level, all of which exhibited marginal main effects. Additionally, we replicated 432 previously reported AD-related genes and found 11 gene–gene interaction pairs overlapping with the protein-protein interaction (PPI) network. Our findings may contribute to partially explain the “missing heritability” of AD. The identified subnetwork may be associated with synaptic dysfunction, Wnt signaling pathway, oligodendrocytes, inflammation, hippocampus, and neuronal cells.

## Introduction

Alzheimer’s disease (AD) is a progressive neurodegenerative disorder characterized by cognitive decline involving memory loss, judgment, reasoning, and impaired daily functioning. It is the most common cause of dementia among the elderly worldwide. The exact etiology of AD is not fully understood, especially the genetic mechanisms of AD, but it is believed to result from complex interactions between genetic and environmental factors^1–5^. For the last two decades, genome-wide association studies (GWAS) have been a successful approach of AD for exploring the correlation between different gene variations and different phenotypic changes. However, the traditional GWAS is mainly based on the association study of single-nucleotide polymorphisms (SNPs) with main effects in the whole genome, and these results only explained part of the “missing heritability” in AD^6,7^.

The “missing heritability” refers to the phenomenon where the heritability of a trait or disease cannot be entirely explained by the genetic variants identified through traditional GWAS. It suggests that there are additional genetic factors contributing to the trait or disease that have not been discovered yet^8–10^.

Epistatic interactions, also known as gene–gene interactions, refer to the phenomenon in genetics where the effect of one gene mask or modifies the effect of another gene. These interactions can influence the expression, phenotype, or inheritance patterns of traits^8,11–13^. Epistasis detection has attracted much attention in recent years, because mounting evidence has shown that epistatic interactions might partially explain the “missing heritability” of AD and will help us to better understand the genetic architecture that underlies complex variation of phenotypes for complex diseases such as AD^14,15^.

However, detecting epistatic interactions across the whole genome still remain twofold challenges of statistical and heavy computational burden. To combat these challenges, many machine learning methods and multifactor dimensionality reduction (MDR) methods were performed, but the results identified by which were challenged for “missing heritability”^16,17^. Furthermore, most of these methods are based on case-control designs, with limited research focusing on performing epistatic interactions detection for quantitative trait (QT)^18,19^.

Although the aforementioned methods have demonstrated their effectiveness on identifying epistasis from genome-wide data, the results identified by which were still challenged for “missing heritability”. One possible reason is that those methods typically used screening strategy, which were easy to missed some SNPs with marginal main effects. Another reason may be the advantages of QT over case–control. To our knowledge, QT analysis allows for the use of continuous measurements, providing more information and increasing statistical power compared to dichotomous outcomes used in case-control studies. In the capture of variation, QT capture the full spectrum of variation within a population, allowing for a more comprehensive understanding of genetic influences on complex traits. At the same time, QT analysis enables the examination of subtle differences in phenotype among individuals, facilitating the identification of marginal effect sizes and complex genetic interactions. Noteworthy, QT can represent intermediate phenotypes or endophenotypes, which may be closer to underlying biological mechanisms and provide insights into disease pathways^8,20–22^.

In recent years, the use of the cerebrospinal fluid (CSF) phosphorylated tau (P-tau)/amyloid-$\beta _{42}$ (A$\beta _{42}$) ratio was an important intermediate endophenotype in AD research, which enabling the understanding of disease progression, diagnosis, and differentiation from other neurodegenerative disorders. It’s worth noting that there are many more studies available on this topic, and further research continues to explore the significance of these biomarkers in AD. The researchers found that this ratio was significantly higher in AD compared to frontotemporal dementia (FTD), suggesting its potential utility as a diagnostic biomarker^23–26^.

Overall, this paper aims to detect the genome-wide SNP–SNP interaction by using P-tau/A$\beta _{42}$ ratio as QT, without disregarding the possibility of SNP-SNP interactions between the marginal main effects, which may be the crucial way for explaining the “missing heritability” of AD. In addition, a muti-GPU method was used for parallel computing to enhance the detection power of SNP–SNP interaction.

## Results

### Two-marker Interaction Study Results and Post Hoc Analysis

**Figure 1:**
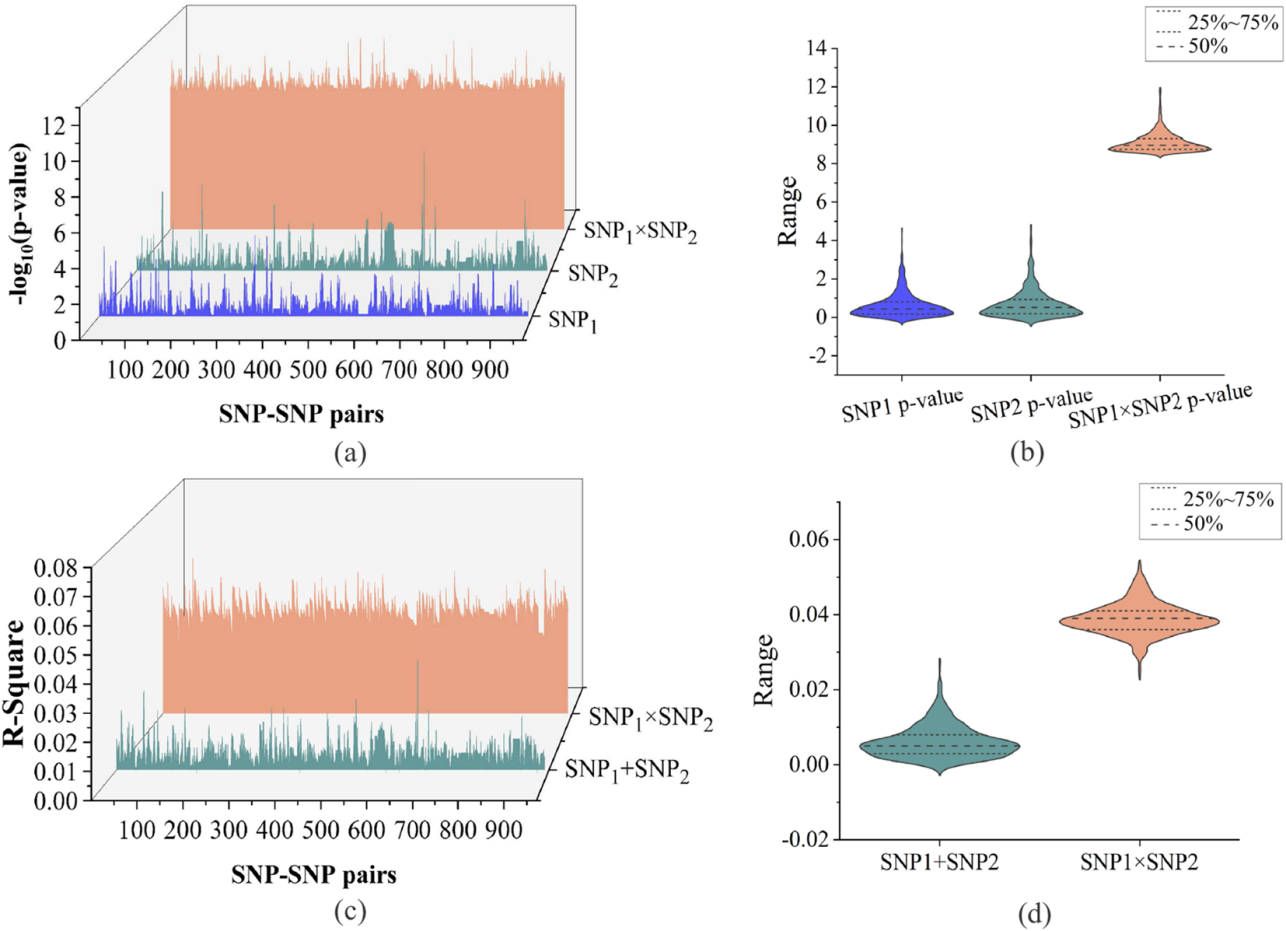
The main effect and interaction effect of 961 SNP-SNP interaction pairs at P-tau/A$\beta _{42}$ ratio level, and R square of the additive terms and the interaction terms. (**a**) The waterfall comparison plot of interaction effects and main effects of single SNP with −log10 adjusted *p*-value. The bule waterfall represents main effects of SNP_1_, the green waterfall represents main effects of SNP_2_, the orange waterfall represents the interaction effects of SNP_1_ $\times$SNP_2_. (**b**) The violin distribution of interaction effects (SNP_1_ $\times$SNP_2_) data and main effects (SNP_1_, SNP_2_) data. (**c**) The waterfall comparison plot of the R square of SNP_1_ $\times$SNP_2_ term (orange waterfall) and the R square of SNP_1_ + SNP_2_ term (green waterfall). (**d**) The violin distribution of R square: interaction (SNP_1_ $\times$SNP_2_) and additive terms (SNP_1_ + SNP_2_).

We used age, gender, and clinical diagnosis (Dx) as covariates in our two-marker interaction study. A total of 1469 SNPs (961 pairs of SNP-SNP interaction) passed the *p*-value threshold at P-tau/A$\beta _{42}$ ratio level. Linkage disequilibrium was assessed by querying the 961 pairs of SNP-SNP interaction using PLINK v1.90 and Haploview 4.1 tool with R^2^ threshold of 0.8. The results indicated that these SNPs are considered to be largely independent (see Supplementary Table S1 online). Considering the technical complexities of pairwise scan of SNP-SNP interaction across the whole genome, we used a muti-GPU tool named GEEpiQt to combat with the heavy computational burden^27^. In addition, the genotype data and participants information of the corresponding loci were extracted according to the SNP-SNP interaction combination information with significant interaction. The main effect of each SNP site was obtained by GWAS, which was used to compare with two-marker interaction results. In this paper, the main effect and interaction effect of 961 SNP-SNP interaction pairs at P-tau/A$\beta _{42}$ ratio level are statistically compared (Fig. 1a). The violin plot shows the numerical distribution and corresponding probability density of the main effect and interaction effect of SNP_1_ and SNP_2_ (Fig. 1b). As show in Fig. 1a, the blue and green areas respectively represent the main effects of SNP_1_ and SNP_2_ in the interaction combination, and the red part represents the interaction effect after the interaction of SNP_1_ and SNP_2_. Moreover, from the overall distribution, the average distribution of interaction effect was much higher than the distribution level of main effect. It suggests that the statistical significance of the interaction effect was much higher than the main effect. The results also showed that there was a significant interaction between SNPs with marginal main effect in terms of statistical implications.

The top 10 R-squared values of SNP–SNP interaction pairs are shown in Table 1. The results of top 10 two-marker interaction were: rs2223610(*MROH9*)–rs12684467, rs11082417(*SETBP1*)–rs1966778(*PITPNM3*), rs17810546(*IL12A-AS1*)–rs17836920(*LINC02074*), rs9493790(*TBPL1*)–rs7955904(*SSPN*), rs17809756(*IL12A-AS1*)–rs17836920(*LINC02074*), rs9730519–rs12458881(*NEDD4L*), rs718222 (*PRICKLE2*)–rs437202(*CSMD1*), rs7617057(*GXYLT2*)–rs17051925(*PARP4P2*), rs3860070(*KLB*)–rs6912680(*ANKRD6*), rs1780555(*RAP1A*)–rs7074813(*RAB18*).

**Table 1:** Top 10 R square of SNP–SNP interaction pairs of the P-tau/A$\beta _{42}$ ratio.

No	SNP–SNP	CHR	Gene	*p*-value		Explained variance (R square)		
				GWAS	Interaction	Age+gender+Dx^a^	SNP_1_+SNP_2_^b^	SNP_1_ $\times$SNP_2_^c^
1	rs2223610 rs12684467	1 9	*MROH9* –	0.496 0.281	1.20E−11	0.133	0.011	0.058
2	rs11082417 rs1966778	18 17	***SETBP1*** ***PITPNM3***	0.025 0.061	3.08E−10	0.133	0.002	0.054
3	rs17810546 rs17836920	3 17	*IL12A-AS1* *LINC02074*	0.087 0.169	1.25E−12	0.133	0.006	0.053
4	rs9493790 rs7955904	6 12	***TBPL1*** ***SSPN***	0.188 0.082	1.09E−9	0.133	0.003	0.053
5	rs17809756 rs17836920	3 17	*IL12A-AS1* *LINC02074*	0.259 0.169	1.75E−12	0.133	0.006	0.052
6	rs9730519 rs12458881	1 18	– ***NEDD4L***	0.272 0.482	8.59E−11	0.133	0.009	0.051
7	rs718222 rs4372022	3 8	***PRICKLE2*** ***CSMD1***	0.708 0.096	2.50E−09	0.133	0.006	0.051
8	rs7617057 rs17051925	3 13	**GXYLT2** *PARP4P2*	0.117 0.004	2.12E−12	0.133	0.001	0.050
9	rs3860070 rs6912680	4 6	*KLB* *ANKRD6*	0.981 0.082	2.33E−11	0.133	0.018	0.050
10	rs1780555 rs7074813	1 10	***RAP1A*** ***RAB18***	0.072 0.378	3.57E−10	0.133	0.005	0.050

^a^ Age+gender+Dx: the percent of variance in P-tau/A$\beta _{42}$ ratio level explained by age, gender, and Dx.

^b^ SNP_1_+SNP_2_: in addition to accounting for three covariates of age, gender, and Dx, the percent of additional variance P-tau/A$\beta _{42}$ ratio level explained by the combined main effect of SNP_1_ and SNP_2_.

^c^SNP_1_ $\times$SNP_2_: In addition to accounting for age, gender, and Dx, SNP_1_ and SNP_2_, the percent of additional variance in P-tau/A$\beta _{42}$ ratio level explained by the interaction effect of SNP_1_ and SNP_2_.

Furthermore, Table 1 also shows the SNP-SNP interaction results of post hoc analysis on P-tau/A$\beta _{42}$ ratio level, and the additive term of SNP_1_+ SNP_2_, the interaction term of SNP_1_ $\times$SNP_2_ and the explained variance of covariates are listed in detail. In this paper, IBM SPSS 24.0 was used to explain variance of SNP-SNP pair with significant interaction on P-tau/A$\beta _{42}$ ratio. The R square of the additive terms (SNP_1_+ SNP_2_) and the interaction terms (SNP_1_ $\times$SNP_2_)are shown in Fig. 1c. The orange area and the green area represent the variance explained by interaction term and additive term at the P-tau/A$\beta _{42}$ ratio level respectively. In order to further obtain the overall distribution of R-square, the distribution state and corresponding probability density of R-square with additive term and interactive term are shown in Fig. 1d.

As shown in Table 1, all covariates (include age, gender, and Dx) accounted for 13.3% of variance on the P-tau/A$\beta _{42}$ level. The variance related to interaction term is determined by running a hierarchical linear regression model, that is, adding genetic main effect first and then adding genetic interaction term. Intriguingly, the variance proportion of the interaction term is much higher than the main effects for each identified SNP-SNP interaction pair as seen from the Table 1. As shown by the top three SNP pairs in the Table 1, the main effects of SNPs accounted for 1.1% of variance, and the interaction term accounted for 5.8% of variance, that is, the combination of the two accounts for 6.9% for the first-ranked rs2223610(*MROH9*)–rs12684467. And for the second-ranked rs11082417(*SETBP1*)–rs1966778(*PITPNM3*), the main effects accounted for 0.2% of variance, and the interaction term accounted for 5.4% of variance, that is, the combination of the two accounts for 5.6%. For the third-ranked rs17810546(*IL12A-AS1*)–rs17836920(*LINC02074*), the main effects accounted for 0.6% of variance, and the interaction term accounted for 5.3% of variance, that is, the combination of the two accounts for 5.9%. Our study revealed that the main effects of the identified interaction SNP pairs are all marginal, but they explained a relatively high-level of variance of P-tau/A$\beta _{42}$ ratio. The gene marked in bold indicates that it is directly related to AD.

### Gene set enrichment analysis results

For illustrating the correlation between the SNP-SNP interaction pairs and AD from different angles and using different databases, a total of 961 SNP-SNP interaction pairs were mapped onto 286 gene–gene pairs (include 505 different genes) by Homo sapiens genome assembly *GRCh37*. First, by searching in two gene set libraries of Enrichr: PhenGenI Association 2021 and HDSigDB Human 2021, we found that 432 genes out of 505 genes were included in the items related to AD, and 85 genes were not included in the items related to AD, which indicated that 432 genes were reported as AD-related genes, and 85 genes were not yet reported as AD-related genes.

**Figure 2:**
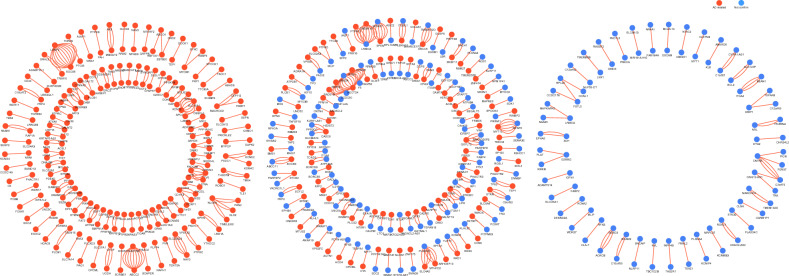
The circle diagram of gene–gene interaction pairs based on the Enrichr database: shows three relationships between genes and AD. All genes related to AD are shown in red dots. The genes that not yet been reported as AD-related are shown in bule dots. G1 include 122 pairs of gene–gene interaction (221 different genes); G2 include 117 pairs of gene–gene interaction (225 different genes); G3 include 47 pairs of gene–gene interaction (85 different genes).

Given that two genes in a gene–gene interaction pair have three relationships with AD: both genes are related to AD, only one gene is related to AD, and neither gene of the two in a gene–gene pair is related to AD, so we divide these interaction pairs into three groups (G1, G2, and G3), as shown in Fig. 2. Specifically, G1 represents all gene pairs in this group that have entries containing the keyword “AD”. We consider these gene pairs to be reported as associated with AD. G2 represents gene pairs in this group where only one gene has an entry containing the keyword “AD”. G3 represents gene pairs in this group where neither gene has an entry containing the keyword “AD”, indicating that these genes have not been reported as associated with AD.

**Figure 3:**
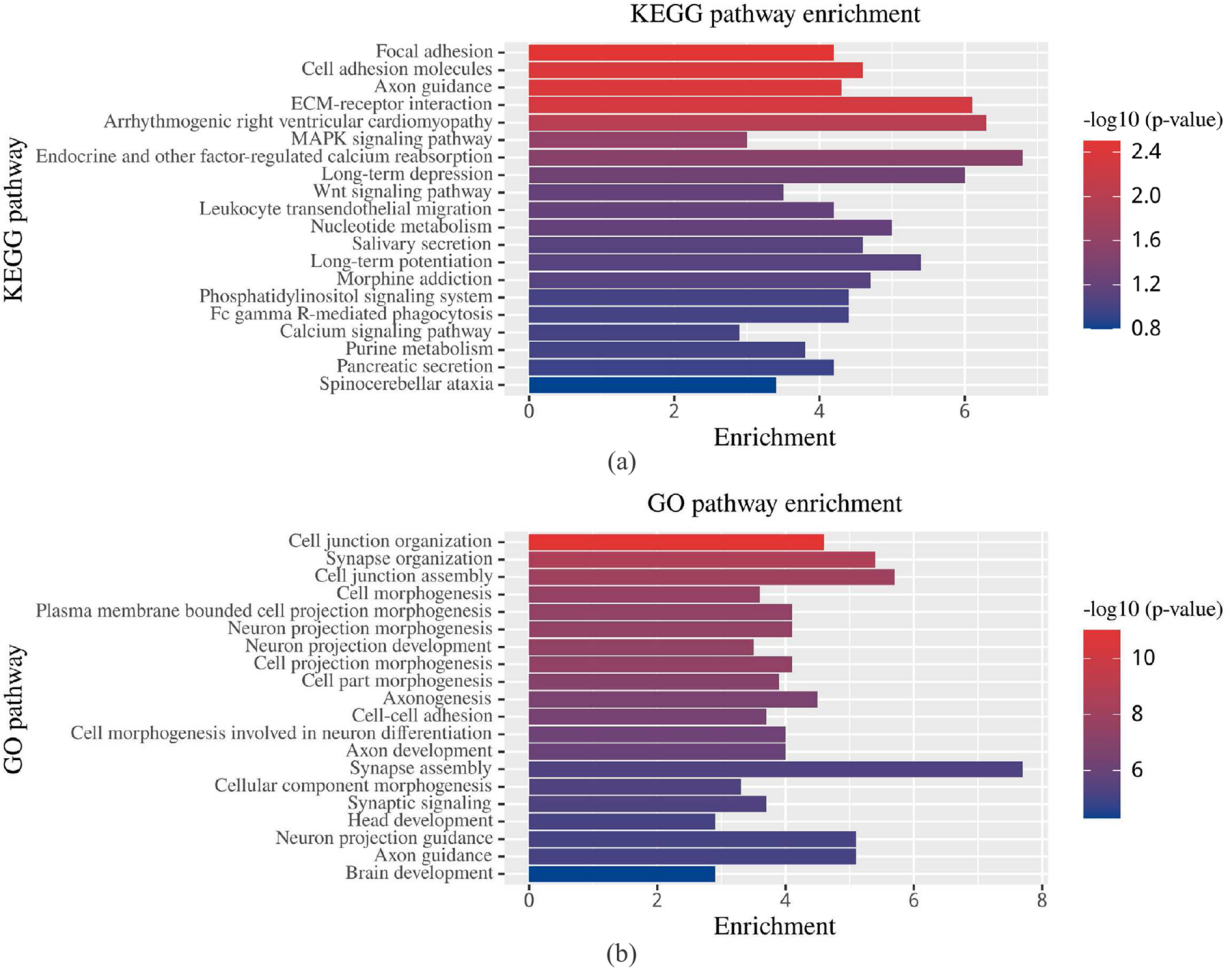
Results of KEGG pathway enrichment analysis and GO functional enrichment analysis. (**a**) Enriched KEGG terms ranked top 20 based on −log10 adjusted *p*-values. (**b**) Enriched GO terms ranked top 20 based on −log10 adjusted *p*-values. Numbers of genes contributing to each term is displayed as bars.

In addition, the biological significance of our results was analyzed further based on two well-known biological pathway databases including KEGG (Kyoto Encyclopedia of Genes and Genomes) and GO (Gene Ontology)^28–30^. The top 20 biological processes based on the corresponding *p*-value of KEGG/GO are shown in Fig. 3.

The bar plot of KEGG pathway enrichment analysis showed that the significant biological processes include focal adhesion (FA), cell adhesion molecules, axon guidance, Wnt signaling pathway, and calcium signaling pathway, etc^31–34^. Foremost among these, As shown in Fig. 3a the most significant biological process was FA, which may be an important participator in the AD process. Moreover, FA signaling might regulate neuronal viability and synaptic loss during AD^35,36^.

Using GO functional enrichment analysis, we found that the significant pathway enrichments were related to AD, such as cell junction organization, synapse organization, cell junction assembly, neuron projection morphogenesis, neuron projectionc development, and cell–cell adhesion^37–41^. As aforementioned, the analysis of function and pathway enrichments improve the biological interpretability of our results.

### Network analysis results

**Figure 4:**
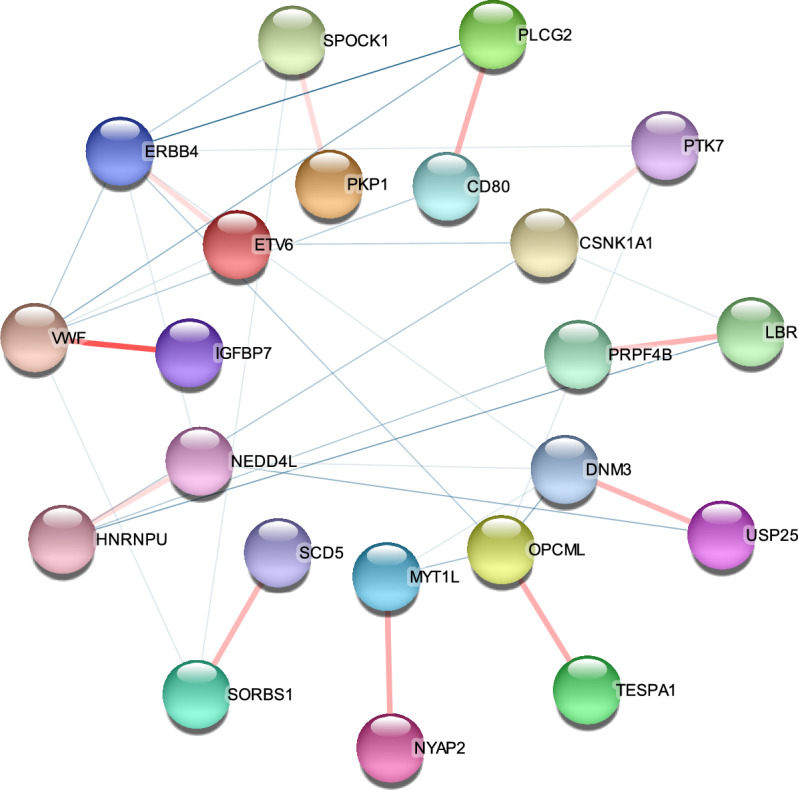
Plot of PPI network with 22 nodes and 11 edges construct by Cytoscape software. The nodes represent the identified genes (protein), and the pink edges represent the overlapped gene–gene interactions.

It is noticeable that protein–protein interaction (PPI) networks provide valuable information for predicting genotype-phenotype associations. They aid in understanding the relationship between genetic variations and phenotypic traits. Therefore, we used 286 pairs of gene–gene interaction from G1, G2 and G3 data sets for PPI network analysis to further interpret biological significance of our results. As shown in Fig. 4, 11 pairs of gene–gene interaction were identified to be overlapping with the PPI network by using the STRING tool (https://string-db.org/, Version 12, accessed on 31 July 2023). Nodes and edges in the PPI network represent the genes and interactions between two genes, respectively. The 22 nodes respectively represent 22 genes, which are: *CSNK1A1*, *PTK7*, *HNRNPU*, *NEDD4*, *CD80*, *PLCG2*, *IGFBP7*, *VWF*, *ERBB4*, *ETV6*, *DNM3*, *USP25* and *MYT1L*, *NYAP2*, *TESPA1*, *OPCML*, *SCD5*, *SORBS1*, *LBR*, *PRPF4B*, *PKP1*, and *SPOCK1*. The interactions between them warrant further discussion in terms of biological implications.

## Discussion

This is a first genome-wide SNP-SNP interaction study using P-tau/A$\beta _{42}$ ratio as an AD imaging QT based on linear regression framework. Our study found 961 SNP–SNP interaction pairs to be statistically significant. Additionally, all these interaction pairs had marginal main effects and explained a relatively high-level variance at the P-tau/A$\beta _{42}$ ratio level. Two gene set libraries of Enrichr: HDSigDB Human 2021 and PhenGenI Association 2021 showed that 320 genes were reported as AD-related genes after mapping the identified SNPs onto the genome. Further gene set enrichment analyses showed that the significant pathway enrichments are associated with AD. These findings are in line with AD pathology and maybe contributes to partially explain the “missing heritability” of AD. Importantly, our results overlapped 11 pairs of gene–gene interaction with the PPI network, which are *CSNK1A1–PTK7*, *HNRNPU–NEDD4*, *CD80–PLCG2*, *IGFBP7–VWF*, *ERBB4–ETV6*, *DNM3–USP25*, *MYT1L–NYAP2*, *TESPA1–OPCML*, *SCD5–SORBS1*, *LBR–PRPF4B*, and *PKP1–SPOCK1*, as shown in Fig. 4. Further analysis of the biological significance was as follows.

Casein kinase I isoform alpha (*CSNK1A1*) can phosphorylate many proteins and participates in Wnt signaling. *PTK7* is a versatile receptor and interact with Wnt signaling pathways. To our knowledge, synapse loss is the prominent event in early stages of AD, and the pathology of AD is amyloid-$\beta _{42}$ (A$\beta _{42}$), and total and phosphorylated tau (T-tau and P-tau). Intriguingly, disrupted Wnt signaling may be a direct link between A$\beta$-toxicity and tau hyperphosphorylation. In parallel, A$\beta$ may trigger the deregulation of Wnt signaling, which could contribute to impaired synaptic plasticity and synapse dysfunction. It suggests that the deregulation of Wnt pathway is associated with AD, and a sustained loss of Wnt signaling function might control AD pathogenesis. Therefore, the *CSNK1A1–PTK7* interaction may be related to Wnt signaling pathways and synaptic pathology in AD, ultimately promoting the progression of AD^42–46^.

As the largest among the HNRNP proteins, *HNRNPU* has been identified as an RNA binding protein to promote RNA stability and regulate gene transcription. Researchers have noted that MiR-132-3p may alleviates the impairment of learning and memory abilities of AD patients by negatively regulating *HNRNPU* stabilized BACE1^47,48^.

LncRNA nuclear enriched abundant transcript 1 (*NEAT1*) interacts with *NEDD4L* and promote *PTEN*-induced putative kinase 1 (*PINK1*)’s degradation, thereby the interaction between them is assumed to be responsible for mitochondria dysfunction and cognitive impairment. Furthermore, mitochondrial dysfunctions are extremely important for the onset and development of AD pathology^49^. Therefore, *HNRNPU–NEDD4L* interaction might has effects on the impairment of cognitive and memory abilities in AD.

Although the role of phospholipase C-gamma-2 (*PLCG2*) in the pathogenesis of AD is only poorly understood, many genetic studies have shown *PLCG2* plays an important role in late-onset AD pathophysiology and in the neural immune response. An increasing body of evidence suggest that due to the driving effect of microglia, *PLCG2* has higher expression levels in brain regions of late-onset AD patients, and is expressed in human and mouse brain microglia. Moreover, the expression of *PLCG2* is maintained in microglia near plaques in the cerebral tissue of an APP mouse model, and is significantly positively correlated with the density of amyloid plaques. These findings suggest that *PLCG2* is associated with the inflammatory response and is induced by amyloid plaques in AD^50–52^. Human immunomodulatory ligand B7-1 (*CD80*) is a canonical costimulatory molecule, and might mediates synaptic remodeling. Microglia or macrophages lead to the upregulation of *CD80*, which is related to aging, central nervous system inflammation, injury, and neurodegenerative diseases^53^. Therefore, the interaction between *CD80* and *PLCG2* maybe associated with both microglia and inflammatory.

*USP25* gene is a critical regulator of AD pathology, and genetic deletion of *USP25* reduced amyloid deposition^54^. *DNM3* is in neuronal tissues, and mostly expressed in the brain. *DNM3* gene has been previously associated with AD pathology based on an independent proteomic study^55,56^. Therefore, the interaction between *DNM3* and *USP25* maybe associated with AD pathology indirectly.

*MYT1L* is a critical mediator of induced neuron cell reprogramming, which can convert into cholinergic neuronal cells. The cholinergic deficit in the brain is involved in the pathological process of cognitive dysfunction, and may be related to cognitive decline in AD^57^. As a phosphoprotein family, *NYAP* is composed of *NYAP1*, *NYAP2*, and *Myosin16/NYAP3*. The *NYAPs* are expressed predominantly in developing neurons, and controlled remodeling of the actin cytoskeleton^58^. Previous reports have shown an impairment in actin cytoskeleton dynamics can contribute to AD pathology and underlie the synaptic failure in AD. Indeed, synapse loss is an early event in the process of AD and is associated with cognitive decline^59^. Therefore, the *MYT1L–NYAP2* interaction maybe related to the pathogenesis of AD indirectly, and might associate with both neuronal cells and cognitive decline in AD.

*IGFBP7* is a critical regulator of AD-like memory consolidation^60^. Von Willebrand antigen 2 (*VWF*) is the von Willebrand factor. It suggests that *VWF* may contribute to A$\beta$ cerebrovascular deposition, thus associated with pathogenesis of AD indirectly^61^. Therefore, both *IGFBP7* and *VWF* genes have been implicated in AD.

*ERBB4* gene mediates A$\beta$-induced neuropathology through pathway associated with tau^62^. *ETV6* gene is a regulator of AD, which maybe involved in cellular senescence^63^.

The stearoylCoA desaturase-5 (*SCD5*) gene participates in the regulation of neuronal cell growth and differentiation, and may be a molecular link signaling and those neurodegenerative conditions^64^. The *SORBS1* gene is a common gene for cognition changing and has potential function in cognitive impairment. *SORBS1* expression has been found to be upregulated in the hippocampus of AD patients^65^.

*OPCML* gene associated with specific hippocampal cell types, which including subtypes of neurons and glial cells, and plays an important role in the development of AD-related pathology^66^. *TESPA1* is involved in the Ca^2+^ transfer and genetic variation in *TESPA1* might influence the aging process. Mounting evidence has shown that the accumulation of mitochondrial Ca^2+^ is significantly related to neuronal apoptosis and other pathways, and enhanced neuronal apoptosis can lead to the neurodegeneration in AD^67,68^.

The oligomeric tau (oTau) is the key toxic species in tauopathy and can elicit disruption of the nucleocytoplasmic interface which is greatly disrupted in AD. Noteworthy, LaminB2 and lamin B Receptor (*LBR*) is co-localized with oTau. Thus, it suggests that *LBR* gene may be related to AD^69^. The pre-mRNA processing factor 4 homolog B (*PRPF4B*) is a kinase involved in mRNA splicing. The current findings suggest that Loss of function of homeodomain-interacting protein kinase 2 (*HIPK2*) has been linked to the development of Alzheimer’s disease, and the homeodomain-interacting protein kinases (HIPKs) reside on a branch together with *PRPF4B*. However, *PRPF4B* has not yet been reported as AD-related gene^70^.

The Kazal-like domains proteoglycan 1 (*SPOCK1*) endocytic pathway modulates by A$\beta$ precursor protein APP may be related to the accumulation of A$\beta _{40}$ and A$\beta _{42}$ in the cerebrospinal fluid and brain tissue in patients with AD^71^. Mitochondrial autophagy or mitophagy is a quality control mechanism that culls defective mitochondria to prevent cellular damage and dysfunction. Mitochondrial dysfunction underlies many age-related human pathologies, such as AD, Parkinson’s disease (PD) and type II diabetes. Although the knockout of both mitochondrial kinases *PKP1* gene and *PKP2* gene (encoding plakophilin 1 and 2) were found to be related with the mitophagy trafficking, the correlation between *PKP1* and AD has not yet been reported^72^.

Taken together, five pairs of gene–gene interactions showed strong association with AD: *CSNK1A1–PTK7*, *HNRNPU–NEDD4*, *CD80–PLCG2*, *IGFBP7–VWF*, and *ERBB4–ETV6*. Among them the *CSNK1A1–PTK7* interaction maybe related to the synapse dysfunction and Wnt signaling pathways, the *HNRNPU–NEDD4L* interaction might has an impact on the impairment of cognitive and memory abilities in AD, and the *CD80–PLCG2* interaction maybe associated with both microglia and inflammatory. Two pairs of gene–gene interactions maybe related to the pathogenesis of AD indirectly: *DNM3–USP25* and *MYT1L–NYAP2*. And the other two pairs of gene–gene interactions may be related to hippocampus, neuronal cells and cognitive impairment: *TESPA1–OPCML* and *SCD5–SORBS1*. Noteworthy, the possible mechanisms behind *LBR–PRPF4B* and *PKP1–SPOCK1* interactions warrant further investigation.

## Materials and methods

### Subjects

The phenotyping data and genotyping data used in the preparation of this article were obtained from the Alzheimer’s Disease Neuroimaging Initiative (ADNI) database. The genotyping data were collected using two arrays of the Illumina 2.5M array and the Illumina OmniQuad array from the ADNI-1, ADNI-GO, and ADNI-2 cohorts. It should be noted that ADNI-1, ADNI-GO, and ADNI-2 are the first three of the four stages contained in ADNI, and the last stage is ADNI-3. The subjects included 1178 individuals, including 639 males and 539 females, and obtained 687,414 SNP after genotyping. In this study, stringent Quality control (QC) was performed using the PLINK v.1.9 software, and SNPs that do not satisfy the criteria of QC will be removed. The QC procedures for genotyping data is mainly composed of eight steps, including call rate check per SNPs, call rate check per subject, the minimum allele frequencies, gender check, pedigree samples identification, Hardy-Weinberg equilibrium test, population stratification, and chromosome removal. Therefore, strict QC procedures were followed:

- call rate per SNP $\geqslant 95\%$;
- call rate check per subject $\geqslant 95\%$;
- minor allele frequency $\geqslant 5\%$;
- gender check followed the F values: if the F value is less than 0.2, the sample is judged to be female, and if greater than 0.8, it is predicted to be male;
- individuals from the same descent were removed;
- Hardy–Weinberg equilibrium test of *p* $\geqslant 10^{-6}$;
- population stratification analysis: excluded 89 subjects from the analysis that were non-Hispanic Caucasians;
- SNPs on chromosome 1–22.

**Figure 5:**
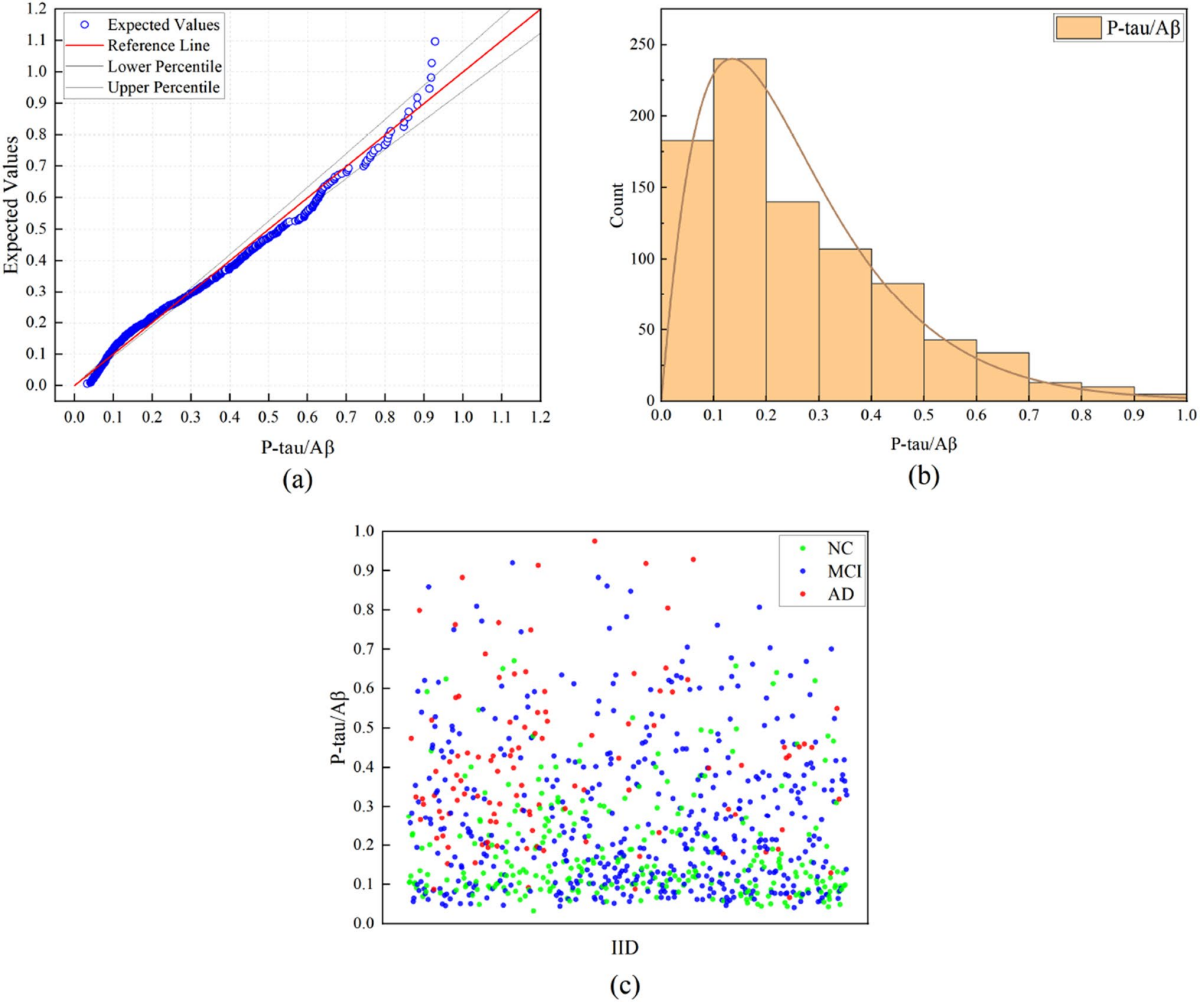
The distribution plot of P-tau/A$\beta _{42}$ ratio after quality control. (**a**) Quantile–quantile (Q–Q) plot of quantitative trait P-tau/A$\beta _{42}$ ratio. (**b**) The bar plot displays the statistical distribution of the quantitative trait P-tau/A$\beta _{42}$ ratio across the number of individuals. (**c**) The scatter plot shows the distribution of the quantitative trait P-tau/A$\beta _{42}$ ratio across the ID of individuals. The red spots represent the ID of Alzheimer’s disease (AD). The blue spots represent the ID of mild cognitive impairment (MCI). The green spots represent the ID of normal controls (NC).

After QC, 858 valid subjects of CSF P-tau/A$\beta _{42}$ ratio remained, and 563,980 SNPs qualified for subsequent genome-wide SNP-SNP interaction analyses. The study cohort comprised 284 normal controls (NC), 459 individuals with mild cognitive impairment (MCI), and 115 AD participants. Table 2 presents the demographic and clinical characteristics of the selected participants at baseline, with detailed data provided in Supplementary Table S2 online. The distribution of the P-tau/A$\beta _{42}$ ratio within our study population is shown in Fig. 5.

**Table 2:** Demographic and clinical characteristics of 858 ADNI participants at baseline analyzed in this study.

Diagnosis	NC (N = 284, 33.1%)	MCI (N = 459, 53.4%)	AD (N = 115, 13.4%)
Age (years)	73.71 (5.80)	71.85 (7.40)	74.92 (8.30)
Women	145 (51.0%)	189 (41.1%)	43 (37.3%)
Education (years)	16.52 (2.61)	16.22 (2.76)	15.70 (2.72)
CDR-SOB	0.05 (0.21)	1.32 (1.00)	3.90 (2.14)
MMSE	27.07 (7.35)	24.88 (8.89)	20.23 (8.11)
CSF A$\beta _{42}$	199.42 (51.75)	173.82 (52.59)	137.27 (35.72)
CSF P-tau	32.8 (16.56)	39.69 (20.86)	53.03 (22.97)
P-tau/A$\beta _{42}$ ratio	0.19 (0.13)	0.27 (0.19)	0.41 (0.21)

Data are number (%) or mean (s.d.).

*AD* Alzheimer’s disease, *ADNI* Alzheimer’s Disease Neuroimaging Initiative, *CDR-SOB* clinical dementia rating-sum of boxes, *CSF* cerebrospinal fluid, *NC* normal controls, *MCI* mild cognitive impairment, *MMSE* Mini-Mental Status Examination.

### Linear detection model of SNP–SNP interaction

This article uses the multiple linear regression model, a statistical model, for interaction detection. Multiple linear regression is a classical statistical learning method widely applied in modeling and prediction of various real-world problems. The model establishes a linear relationship between multiple independent variables and a dependent variable, and estimates the model parameters using the least squares method (LSM). The multiple linear regression model has strong interpretability and predictive performance. Using a linear model to detect SNP interactions allows for intuitive interpretation of the impact of interactions on phenotypes, providing relevant statistics and significance detection results. Additionally, the linear model can handle issues like interaction and collinearity among multiple independent variables, effectively capturing the influence of SNP–SNP interactions on phenotypes.

Moreover, the parameter estimation methods of linear models are relatively simple, using classic optimization algorithms such as LSM, which are computationally efficient and capable of handling large-scale genetic data. Furthermore, these methods facilitate the interpretation of model parameters and statistics. Taking into account the factors mentioned above, this article utilizes multiple linear regression to detect SNP–SNP interactions.

In this model, the dependent variable is predicted based on a linear combination of the independent variables, each multiplied by its respective coefficient. The data in this article consists of m subjects, each having n SNPs and one quantitative trait phenotype. Let S be the genotype matrix of the SNP array, expressed as:$S = ( {{S_1},{S_2}, \ldots ,{S_n}})$, where any SNP genotype vector expressed as: ${S_i} = \left( {{S_{1i}},{S_{2i}}, \ldots ,{S_{mi}}} \right)$, where $S_{ij}=0,1,2$ represents the genotype data of the *i*th individual at the *j*th SNP locus. Let Y be the measured quantitative trait, represented by an M$\times$1 phenotype matrix. The essence of interaction detection is to consider interaction factors based on the main effects of SNPs and calculate the *p*-values of these interaction factors.

It is worth noting that the SNP$\times$SNP model consisted of the same SNP and covariate terms (include age, gender, Dx) as the additive model, while with an additional multiplicative interaction term for the SNP$\times$SNP model. Finally, the *p*-value of the interaction is calculated according to the F statistic.

### Functional enrichment analysis and functional annotation

Functional enrichment analysis is a computational method that determines if a group of genes (proteins) is associated with specific biological functions. Annotation analysis assigns functional information to genes (proteins) based on available databases. When combined, these analyses help researchers understand the biological implications of gene or protein sets by identifying related functions and providing detailed annotations. This approach is widely used in biological studies to gain insights into underlying mechanisms and pathways. In this study, genome annotations and analyses were conducted utilizing two distinct annotation tools: Ensembl (http://www.ensembl.org/) and ANNOVAR (https://annovar.openbioinformatics.org/). The results demonstrated a high level of concordance between the findings obtained from both tools, accompanied by comprehensive gene annotation information presented in Supplementary Table S3 online. For SNPs that could not be directly mapped to genes, we performed gene annotation using Homo sapiens genome assembly GRCh37 (hg19) with a distance threshold of 100 kb. This approach enabled the identification of potential regulatory elements and functional associations in close genomic proximity to the SNPs of interest, facilitating the exploration of their potential biological significance. PhenGenI Association 2021 and HDSigDB Human 2021 of Enrichr libraries were used to discuss the correlation between the identified genes and AD. Our intention was to preliminarily assess the association of two genes in a pair of gene–gene interactions with AD at the genetic level using these two databases. We achieved this by searching for whether these genes contained items related to AD. Specifically, if a gene contained items related to AD, we included it in the group of genes reported as being associated with AD. Genes that did not contain items related to AD were placed in the group of genes not yet reported as being associated with AD. In addition, pathway analyses for KEGG and GO were performed on all results with-log10 adjusted *p*-values. The PPI network plays a crucial role in the prediction of genotype–phenotype associations. As one key role of PPI network, functional annotation can infer the functions of genes and their associated phenotypic features. Therefore, PPI network enrichment analysis was also performed on gene–gene interaction pairs to analyze further their biological implications.

## Conclusion

In this paper, we conducted a genome-wide SNP–SNP interaction analysis based on P-tau/A$\beta _{42}$ ratio. We used a linear regression model with age, gender, and Dx as covariates. To expedite the search speed for SNP–SNP interactions at the whole-genome level, we employed a parallel computing approach based on multiple GPUs. By using P-tau/A$\beta _{42}$ ratio as QT, we increased statistical power and facilitated the identification of SNP–SNP interactions with marginal main effects. We identified 961 SNP–SNP interaction pairs with marginal main effects that contribute to explained a relatively high-level variance of P-tau/A$\beta _{42}$ ratio. Notably, a total of 432 previously reported AD-related genes were replicated, and 11 gene–gene interaction pairs were found to overlap with the PPI network. Nine of these gene–gene interaction pairs were potentially related to synaptic dysfunction, Wnt signaling pathway, oligodendrocytes, inflammation, hippocampus, and neuronal cells, which may ultimately lead to cognitive decline in AD. The potential mechanisms underlying the interactions of *LBR-PRPF4B* and *PKP1-SPOCK1* warrant further investigation. Our findings contribute to the partial elucidation of the “missing heritability” of AD. Future research should involve various methods and datasets to validate and further elucidate the biological significance of our results.

### Supplementary Information

Supplementary Table S1.

Supplementary Table S2.

Supplementary Table S3.

## Data Availability

The data used in this project were funded by the Alzheimer’s Disease Neuroimaging Initiative (ADNI). The database is the (http://adni.loni.usc.edu/, accessed on 1 September 2023) of the Alzheimer’s disease neuroimaging database.
